# Sputum procalcitonin levels in patients admitted to hospital with acute exacerbations of bronchiectasis

**DOI:** 10.1002/hsr2.203

**Published:** 2020-11-27

**Authors:** William Good, Sarah Mooney, Irene Zeng, Susan Taylor, Lata Jayaram, David Holland, Benjamin Diggins, Conroy Wong

**Affiliations:** ^1^ Department of Respiratory Medicine Middlemore Hospital, Counties Manukau District Health Board Auckland New Zealand; ^2^ Department of Medicine, Faculty of Medical and Health Sciences The University of Auckland Auckland New Zealand; ^3^ Research Office, Middlemore Hospital Counties Manukau District Health Board Auckland New Zealand; ^4^ Department of Microbiology Middlemore Hospital, Counties Manukau District Health Board Auckland New Zealand; ^5^ Department of Respiratory Medicine Western Health Melbourne Victoria Australia; ^6^ Melbourne Medical School The University of Melbourne Melbourne Victoria Australia; ^7^ Department of Infectious Diseases Middlemore Hospital, Counties Manukau District Health Board Auckland New Zealand

## Abstract

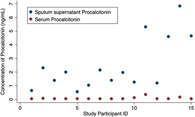

## 1 INTRODUCTION

Bronchiectasis is a chronic, debilitating disease typified by productive cough, airway inflammation and repeated respiratory tract infections. It is characterised by persistent airway neutrophilic inflammation and airway infection is a major driver for neutrophil recruitment.^1^, ^2^ Multiple biomarkers have been developed to investigate ongoing airway inflammation including sputum neutrophil elastase, sputum cell counts and cytokines, serum C‐reactive protein (CRP), erythrocyte sedimentation rate (ESR) and fibrinogen.^3^, ^4^, ^5^

Procalcitonin is a precursor of calcitonin, a hormone with hypocalcaemic effects that is secreted in response to hypercalcaemia and in healthy individuals, procalcitonin is synthesised by the calcitonin gene (CALC‐I).^6^, ^7^ However procalcitonin was found to be a marker of sepsis, prompting significant interest and research into its utility as a biomarker of infection.^8^, ^9^

The use of procalcitonin as a biomarker in bronchiectasis has been limited with small numbers of study participants. These studies have investigated the relationship of serum procalcitonin in stable and unstable patients with bronchiectasis. Serum procalcitonin was found to be low in the majority of patients with bronchiectasis and was not correlated with other systemic inflammatory markers such as CRP.^10^, ^11^, ^12^ We hypothesised that measurement of sputum procalcitonin would more accurately assess airway inflammation or persistent bacterial infection and therefore provide a better airway biomarker for bronchiectasis.

## 2 METHODS

### 2.1 Study design and participants

This exploratory, cross‐sectional study was conducted at Middlemore Hospital, Auckland, New Zealand. Patients were eligible for inclusion in the study if they were at least 18 years of age, had a diagnosis of non‐cystic fibrosis bronchiectasis based on a high‐resolution computed tomography (HRCT) scan of the chest, and had been admitted to hospital with an acute exacerbation of bronchiectasis. Exacerbation was determined by the treating physician and consecutive patients meeting the inclusion criteria were recruited. Patients with underlying allergic bronchopulmonary aspergillosis, cystic fibrosis and primary ciliary dyskinesia were excluded. Patients who had been taking continuous antibiotics for three or more months prior to hospitalization were also excluded. However, those who had antibiotics started for the current exacerbation in the community were included and pre‐admission antibiotic use was recorded. All patients provided written informed consent to participate in the study, which was approved by an independent regional ethics committee, and conducted in accordance with the principles of Good Clinical Practice and the Declaration of Helsinki.

### 2.2 Procedures

Sputum samples were obtained within 48 hours of admission to hospital. Sputum supernatant was obtained from spontaneous sputum samples that were dispersed using dithiothreitol (DTT) and processed according to European Respiratory Society recommendations.^13^, ^14^ Procalcitonin concentrations in sputum supernatant were measured using the Vidas BRAHMS PCT assay (Brahms GmbH, Hennigsdorf, Germany). Sputum samples were cultured for standard respiratory pathogens and tested for a panel of viruses using real‐time reverse transcription polymerase chain reaction (PCR).

Peripheral blood samples for the measurement of procalcitonin concentration, total white cell count (WCC), neutrophil count, CRP concentration and ESR were collected within 48 hours of hospital admission. Serum procalcitonin levels were also measured using the same assay described above.

### 2.3 Study endpoints

Outcomes recorded during the study included the primary endpoint of procalcitonin concentration in spontaneously obtained sputum; secondary endpoints were serum procalcitonin concentration, peripheral blood white cell and neutrophil counts, serum CRP, ESR, sputum bacterial culture and viruses.

### 2.4 Statistical analysis

Serum and sputum procalcitonin levels were summarized using median values with the interquartile range (IQR). Normally distributed continuous variables were reported as mean values with SDs, and categorical variables as counts and proportions. Correlations between serum and sputum procalcitonin levels and WCC, ESR, CRP, virus (PCR) and symptom scores were assessed using Spearman's correlation coefficients. Log transformation analysis was also performed. All statistical tests are two‐tailed, and a *P*‐value of <.05 was considered to be statistically significant. Statistical analyses were performed using SAS v9.2 (SAS Institute Inc., Cary, North Carolina.).

## 3 RESULTS

### 3.1 Patients

A total of 15 patients admitted to Middlemore Hospital between April and December 2010 with an acute exacerbation of bronchiectasis participated in the study (Table 1). Gender was equally distributed between male (47%) and female (53%); the average age was 58 years (SD 20 years) and 7 (47%) were current or ex‐smokers. Inflammatory markers were elevated, with ESR and CRP each being above the normal range in 87% of patients. At the time of hospital admission eight of the patients were receiving antibiotics, eight were already on oral corticosteroids and twelve were regularly using inhaled corticosteroids. Five patients (33%) were culture positive for *Pseudomonas aeruginosa* and two patients were rhinovirus‐positive identified by PCR (Table 1).

**TABLE 1:** Study patient demographics and exacerbation assessment results

	Patients (n = 15)
Age, years	58 ± 20
Female, n (%)	8 (53)
Smoking status, n (%)	
Current smoker	2 (13)
Ex‐smoker	5 (33)
Ethnic origin, n (%)	
European	6 (40)
Polynesian	7 (47)
Maori	2 (13)
Symptom scores^a^	
Wellness	4 (3–5)
Shortness of breath	4 (2–5)
Sputum colour	4 (4‐5)
Sputum volume	3 (3–5)
Sputum culture, n (%)	
Normal flora	9 (60)
*Pseudomonas aeruginosa*	5 (33)
*Strenotrophomonas multiphilia*	1 (7)
Antibiotics prior to admission, n (%)	8 (53)
Biomarker levels	
CRP, mg/L	26 (11‐55)
ESR, mm/h	42 (19‐51)
WCC, ×10^9^/L	9 (7–12)
Serum procalcitonin, ng/mL	0.05 (0.05‐0.08)
Patients with serum procalcitonin ≤0.05 ng/mL, n (%)	11 (73)
Sputum procalcitonin, ng/mL	2.0 (1.2‐4.6)
Medications, n (%)^b^	
Amoxicillin/clavulanic acid IV	7 (47)
Roxithromycin	6 (40)
Cotrimoxazole/ciprofloxacin/gentamicin	2 (13)
Prednisone	8 (53)
Salbutamol inhaler	8 (53)

*Note*: Values are presented as number of patients (%), mean ± SD, or median (interquartile range).

Abbreviations: CRP, C‐reactive protein; ESR, erythrocyte sedimentation rate; IV, intravenous; WCC, white cell count.

a Symptom scores were on a scale from 1 to 5, with higher scores indicating greater symptom severity. For Wellness, a higher score indicates that the patient better.

b Medication description relates to hospital treatment prior to sputum and serum analysis.

### 3.2 Sputum and serum procalcitonin

**FIGURE 1:**
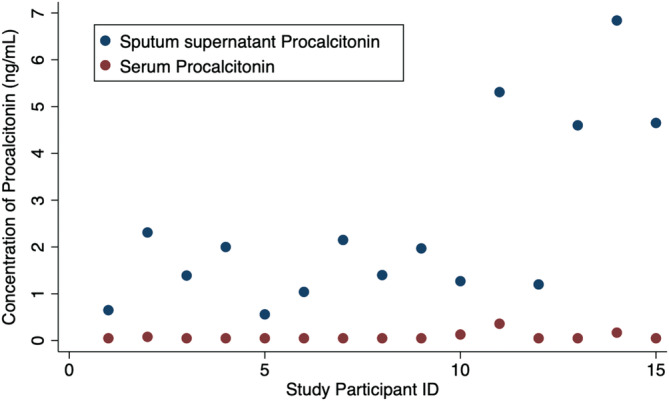
Procalcitonin levels in serum vs sputum supernatant

Procalcitonin levels in sputum supernatant were significantly higher than those in serum (Figure 1). The median sputum procalcitonin level was 2.0 ng/mL (range 0.6‐6.8 ng/mL) while serum procalcitonin ranged from <0.05 to 0.4 ng/mL. All patients had a sputum procalcitonin level of >0.5 ng/mL, whereas only three patients (20%) had a serum procalcitonin level of >0.1 ng/mL, a level used in a procalcitonin‐antibiotic algorithm for lower respiratory tract infections to determine if antibiotics are recommended.^15^ Ten patients had a serum procalcitonin level lower than the lowest quantifiable level of 0.05 ng/mL.

### 3.3 Associations between sputum procalcitonin and inflammatory markers and symptoms

**FIGURE 2:**
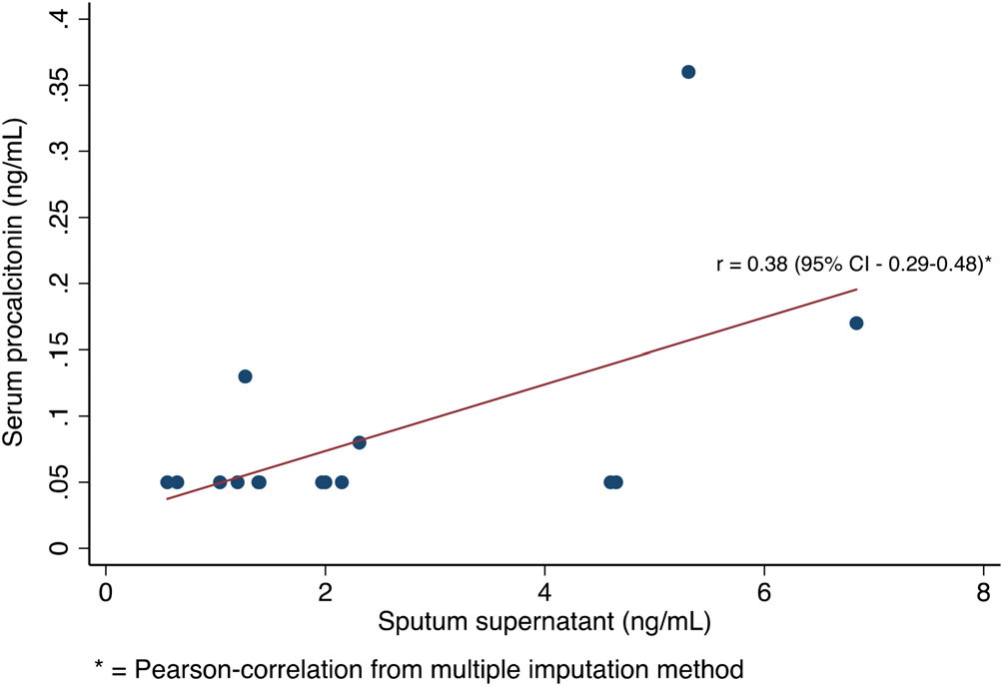
Relationship of serum and sputum procalcitonin

There was a significant positive correlation between baseline sputum procalcitonin concentration and ESR (Spearman correlation coefficient: 0.56, *P* = .04). Sputum procalcitonin did not significantly correlate with CRP but there was moderate positive association (Spearman correlation coefficient 0.37, *P* = .18). Sputum procalcitonin did not correlate with WCC, serum procalcitonin or symptom scores (Table 2). There was no observed difference in sputum procalcitonin concentration relating to positive or negative sputum bacterial culture. It was difficult to determine factors associated with baseline serum procalcitonin because of the high proportion of patients who had levels below the detection limit of the assay (<0.05 ng/mL). However, the median sputum procalcitonin was 1.7 and 2.3 ng/mL in patients with serum procalcitonin <0.05 and ≥0.05 ng/mL respectively. We therefore tried several imputation methods, and ruled out the likelihood that these censored serum procalcitonin will have the same mean as those >0.05 ng/mL. Using a multiple imputation method, we assumed the mean is 0.025 ng/mL and added variation based on the SD of the observed values on log scale (0.33 ng/mL). The mean Pearson‐correlation from multiple imputation was 0.38 (95% CI 0.29‐0.48). This suggests a weak association between serum procalcitonin and sputum procalcitonin (Figure 2).

**TABLE 2:** Associations between sputum procalcitonin and inflammatory markers, sputum procalcitonin and symptoms

	Sputum supernatant procalcitonin
**Correlation with inflammatory markers, *r* (*P*‐value)**	
ESR	0.53 (*P* = .04)
WCC	0.009 (*P* = .97)
CRP	0.37 (*P* = .18)
**Correlation with symptoms, *r* (*P*‐value)**	
Wellness	0.22 (*P* = .42)
Shortness of breath	−0.05 (*P* = .86)
Sputum colour	−0.01 (*P* = .96)
Sputum volume	−0.26 (*P* = .35)

Abbreviations: CRP, C‐reactive protein; ESR, erythrocyte sedimentation rate; IQR, interquartile range; WCC, white cell count.

## 4 DISCUSSION

This exploratory study is the first to investigate sputum rather than serum levels of procalcitonin. The results demonstrate that patients hospitalized with an acute exacerbation of bronchiectasis have substantially higher procalcitonin levels in sputum compared with serum. In addition, sputum procalcitonin is positively associated with ESR.

In this group of study participants with bronchiectasis, two‐thirds had a serum procalcitonin level below the lower detection limit of the Vidas BRAHMS assay at the time of hospital admission for an acute pulmonary exacerbation. This is consistent with the limited literature investigating procalcitonin use in patients with bronchiectasis and our study confirms that serum procalcitonin is not a useful biomarker in infective exacerbations of bronchiectasis.^10^ The relationship of systemic inflammatory markers such as procalcitonin to bronchial inflammation biomarkers remains unclear and the possibility of compartmentalisation within the airways has been proposed.^16^

This study is a proof of concept study evaluating the potential role of sputum procalcitonin testing in patients with bronchiectasis. Procalcitonin has been assessed in other bodily fluids including cerebrospinal fluid (CSF) in patients with meningitis,^17^ pleural fluid in patients with parapneumonic effusions^18^ and bronchoalveolar lavage (BAL) fluid in patients with ventilator‐associated pneumonia.^19^, ^20^ More recently, procalcitonin has been measured in the saliva of patients with Chronic Obstructive Pulmonary Disease (COPD).^21^ This demonstrated that procalcitonin levels in saliva were repeatable over a 14‐day period, increased during acute exacerbations of COPD, and correlated with patient‐derived clinical metrics.

The exploratory nature of this study means that it has a number of limitations. These include a small sample size and ongoing validation requirements of the procalcitonin assay for use with sputum samples. In addition, determination of procalcitonin levels at one time point does not allow for identification of changes in sputum procalcitonin levels over time, in response to therapy, or in relation to patient outcomes. Finally, the influence of prior antibiotic therapy should be acknowledged and its influence on our results are unknown.

In conclusion, we found that sputum procalcitonin levels during acute exacerbations of bronchiectasis were substantially higher than serum levels of this marker. This new sputum biomarker has the potential to help in the assessment and management of bronchiectasis. Further studies to assess the repeatability of sputum testing and how this biomarker changes following an exacerbation and in periods of stability are required.

## CONFLICT OF INTEREST

There is no conflict of interest relating to this study.

## AUTHOR CONTRIBUTIONS

Conceptualization: Associate Professor Conroy Wong

Formal Analysis: Dr Irene Zeng, Dr William Good

Investigation: Dr Susan Taylor

Methodology: Associate Professor Conroy Wong, Dr Sarah Mooney,

Associate Professor Lata Jayaram, Dr David Holland, Dr Susan Taylor

Writing – Original Draft Preparation: Dr William Good, Dr Irene Zeng,

Dr Benjamin Diggins, Associate Professor Conroy Wong

Writing – Review & Editing: Associate Professor Conroy Wong, Dr

William Good, Dr Benjamin Diggins, Dr Irene Zeng, Dr Sarah Mooney,

Associate Professor Lata Jayaram, Dr David Holland, Dr Susan Taylor

 

 All authors have read and approved the final version of the manuscript

 Dr William Good had full access to all of the data in this study and takes complete responsibility for the integrity of the data and the accuracy of the data analysis.

## TRANSPARENCY STATEMENT

*Dr William Good* affirms that this manuscript is an honest, accurate, and transparent account of the study being reported; that no important aspects of the study have been omitted; and that any discrepancies from the study as planned (and, if relevant, registered) have been explained.

## Data Availability

The authors confirm that the data supporting the findings of this study are available within the article. The data that support the findings of this study are available for the corresponding author upon reasonable request.
